# Supplementary material for: On-surface cyclization of vinyl groups on poly-para-phenylene involving an unusual pentagon to hexagon transformation
Source: Nat Commun. 2024 Mar 1;15:1910. doi: 10.1038/s41467-024-46173-3 (PMC10907692; doi:10.1038/s41467-024-46173-3)
Supplement: Supplementary file 1 — Supplementary Information [file 41467_2024_46173_MOESM1_ESM.pdf]

Supplementary Information for

On-Surface Cyclization of Vinyl Groups on Poly-*para*-phenylene Involving An Unusual Pentagon to Hexagon Transformation

Marco Di Giovannantonio,[1,*,#,Ψ] Zijie Qiu,[2,#,±] Carlo A. Pignedoli,[1,#] Sobi Asako,[3,4] Pascal Ruffieux,[1] Klaus Müllen,[2,5,*] Akimitsu Narita,[2,4,*] Roman Fasel[1,6,*]

[1]*Empa, Swiss Federal Laboratories for Materials Science and Technology, nanotech@surfaces Laboratory, 8600 Dübendorf, Switzerland*

[2]*Max Planck Institute for Polymer Research, 55128 Mainz, Germany*

[3]*RIKEN Center for Sustainable Resource Science, Wako, Saitama 351-0198, Japan*

[4]*Organic and Carbon Nanomaterials Unit, Okinawa Institute of Science and Technology Graduate University, Okinawa 904-0495, Japan*

[5]*Department of Chemistry, Johannes Gutenberg University Mainz, Duesbergweg 10-14, 55128 Mainz, Germany*

[6]*Department of Chemistry, Biochemistry and Pharmaceutical Sciences, University of Bern, 3012 Bern, Switzerland*

[#]*These authors contributed equally*

[Ψ]*Present address: Istituto di Struttura della Materia – CNR (ISM-CNR), 00133 Roma, Italy*

[±]*Present address: School of Science and Engineering, Shenzhen Institute of Aggregate Science and Technology, The Chinese University of Hong Kong, Shenzhen, Guangdong 518172, China*

Table of Contents

1. **Precursor synthesis and characterizations**
2. **Additional discussions and experimental/computational results**
3. **Energy barrier estimation**

1. Precursor synthesis and characterizations

General Methods

All reactions working with air- or moisture-sensitive compounds were carried out under nitrogen atmosphere using standard Schlenk line techniques. Unless otherwise noted, all starting materials were purchased from commercial sources and used without further purification. All other reagents were used as received. Preparative column chromatography was performed on silica gel from Merck with a grain size of 0.063–0.200 mm (silica gel). Nuclear Magnetic Resonance (NMR) spectra were recorded in $CDCl_3$ on AVANCE 300 MHz Bruker spectrometers. Abbreviations: s = singlet, d = doublet, t = triplet, m = multiplet, dd = doublet of doublets, br = broad signal. High-resolution mass spectrometry (HRMS) was performed on a SYNAPT G2 Si high-resolution time-of-flight mass (TOF) spectrometer (Waters Corp., Manchester, UK) by matrix-assisted laser desorption/ionization (MALDI) using 7,7,8,8-tetracyanoquinodimethane (TCNQ) as matrix.

Synthesis

1,4-Dibromo-2,5-divinylbenzene (**S2**)[1] and 2,2'-(2,5-divinyl-1,4-phenylene)bis(4,4,5,5-tetramethyl-1,3,2-dioxaborolane) (**S3**)[2] were synthesized and characterized according to the previous reports.

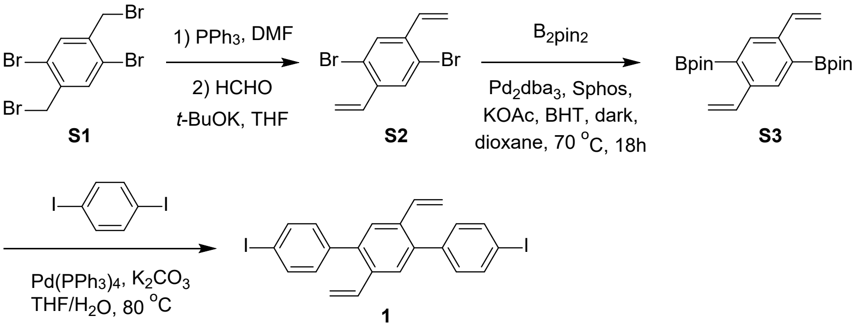

Supplementary Fig. 1 | Synthetic route towards precursor 1. The synthetic steps and conditions that afforded compound **1** are indicated in the reaction sequence.

4,4''-diiodo-2',5'-divinyl-1,1':4',1''-terphenyl (1): Under nitrogen atmosphere, compound **S3** (100 mg, 0.262 mmol), 1,4-diiodobenzene (432 mg, 1.31 mmol, 5 equiv.), $Pd(PPh_3)_4$ (18.1 mg, 0.0157 mmol, 0.06 equiv.), and K_2CO_3 (289 mg, 2.09 mmol, 8 equiv.) were dissolved in a mix solution of 8 mL THF and 4 mL H_2O. Afterwards, the solution was heated to reflux overnight with stirring. After the reaction was completed as monitored by TLC, the solution was extracted with dichloromethane (DCM) for three times. The combined organic phases were dried over magnesium sulfate. After the solvents were removed by rotary evaporation, the residue was purified by silica gel column chromatography (hexane:DCM = 10:1 as eluent), 119 mg of the title compound was obtained as light yellow solid in 85% yield. The precursor **1** was further purified by recrystallization from DCM/MeOH for three times before using for UHV. The NMR analysis of this compound is reported in Supplementary Fig. 10 and 11. ^{1}H NMR (300 MHz, chloroform-*d*, 298 K): δ 7.78 (d, J = 8.3 Hz, 4H), 7.51 (s, 2H), 7.15 (d, J = 8.4 Hz, 4H), 6.72–6.62 (m, 2H), 5.70 (d, J = 17.4 Hz, 2H), 5.22 (d, J = 11.0 Hz, 2H). ^{13}C NMR (75 MHz, chloroform-*d*, 298 K): δ 139.98, 139.31, 137.46, 135.27, 135.01, 131.78, 127.46, 115.76, 93.29. HRMS (MALDI-TOF) *m/z*: Calcd for $C_{22}H_{16}I_2$: 533.9341; Found: 533.9327 (M^+).

2. Additional discussions and experimental/computational results

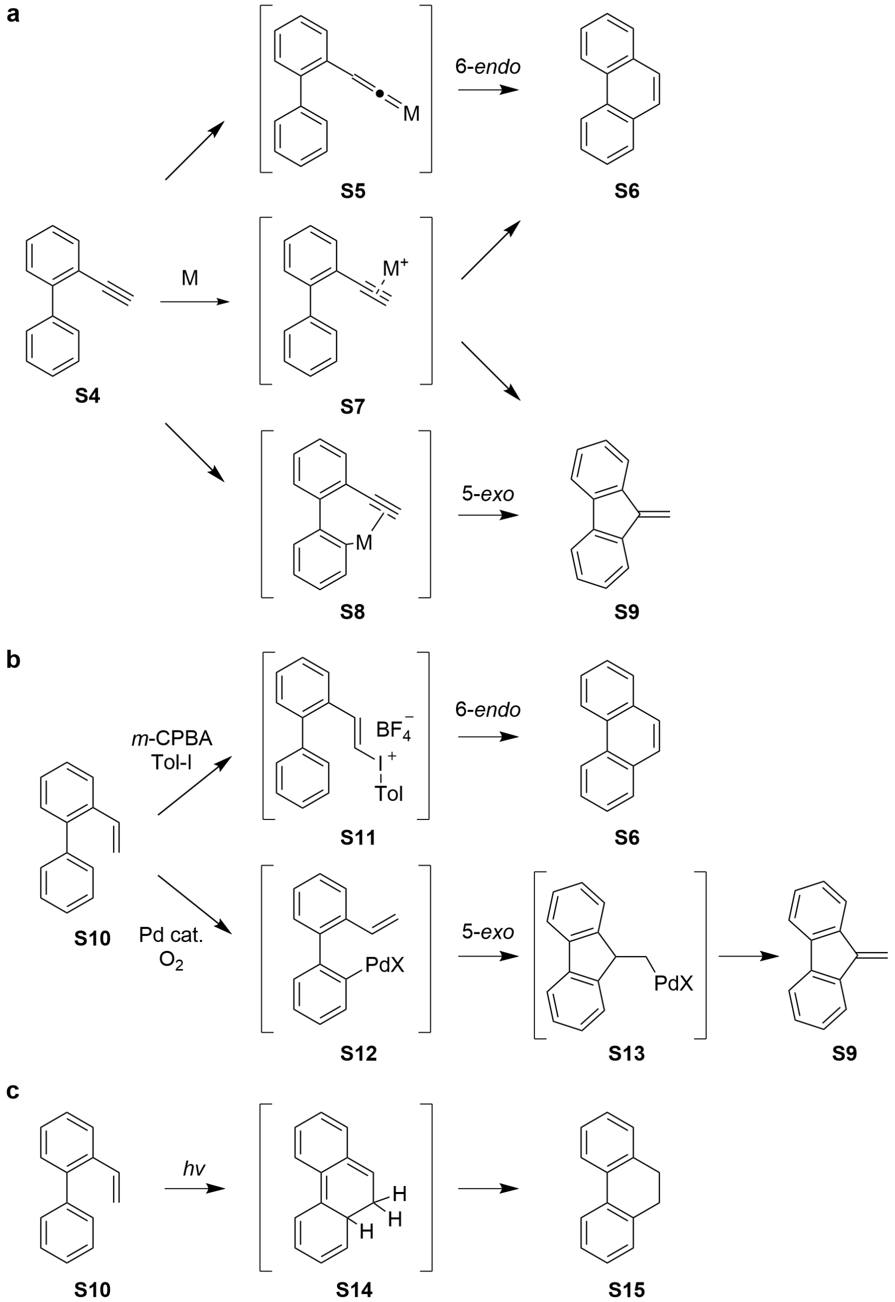

Supplementary Fig. 2 | Overview of similar cyclization reactions reported in solution chemistry processes. a, Intramolecular alkyne arylation of 1-phenyl-2-ethynylbenzene (**S4**).[3] According to the Baldwin's rules, the reaction in **a** can lead to either phenanthrene (**S6**) via a 6-*endo-dig* ring closure or 9-methylene-9*H*-fluorene (**S9**) through a 5-*exo-dig* cyclization. **b,** Phenanthrene (**S6**) or 9-methylene-9*H*-fluorene (**S9**) moieties can be achieved by the hypervalent iodine (HVI)-mediated oxidative cyclization of 2-vinylbiphenyl (**S10**), or the Pd-catalyzed cyclization under aerobic oxidation conditions, respectively.[4] **c**, Photochemical cyclization of 2-vinylbiphenyl (**S10**) in both the absence and the presence of oxygen produces 9,10-dihydrophenanthrene (**S15**) in quantitative yield.[5] Notably, our on-surface reaction experiences a pentagon-to-hexagon transformation, which has never been described in solution chemistry.

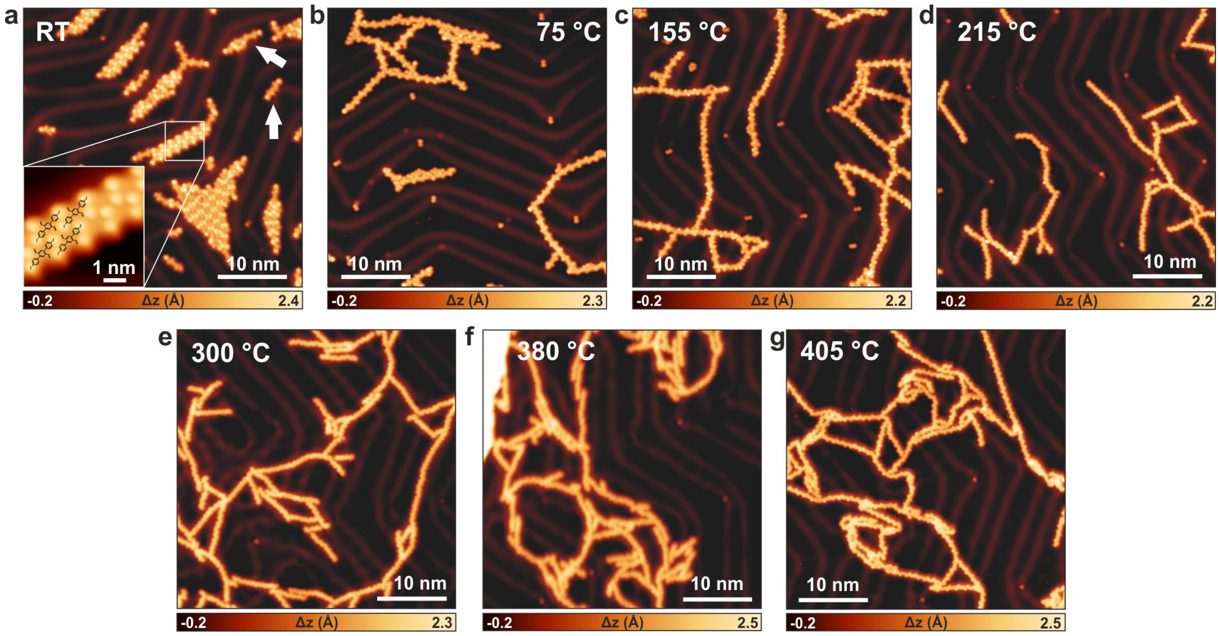

Supplementary Fig. 3 | Large scale STM images after annealing at increasing temperature. White arrows in **a** indicate a dimer and a trimer. The inset shows a zoom-in of a molecular island consisting of intact (non-dehalogenated) molecules. Scanning parameters: **a**, I_t=20 pA, V_b=–1.0 V; **b**, I_t=30 pA, V_b=–1.0 V; **c**, I_t=30 pA, V_b=–0.3 V; **d**, I_t=30 pA, V_b=–0.3 V; **e**, I_t=30 pA, V_b=–0.1 V; **f**, I_t=50 pA, V_b=–0.2 V; **g**, I_t=100 pA, V_b=–0.1 V. Temperatures in each panel indicate the sample annealing temperature, while the imaging was performed at 4.7 K.

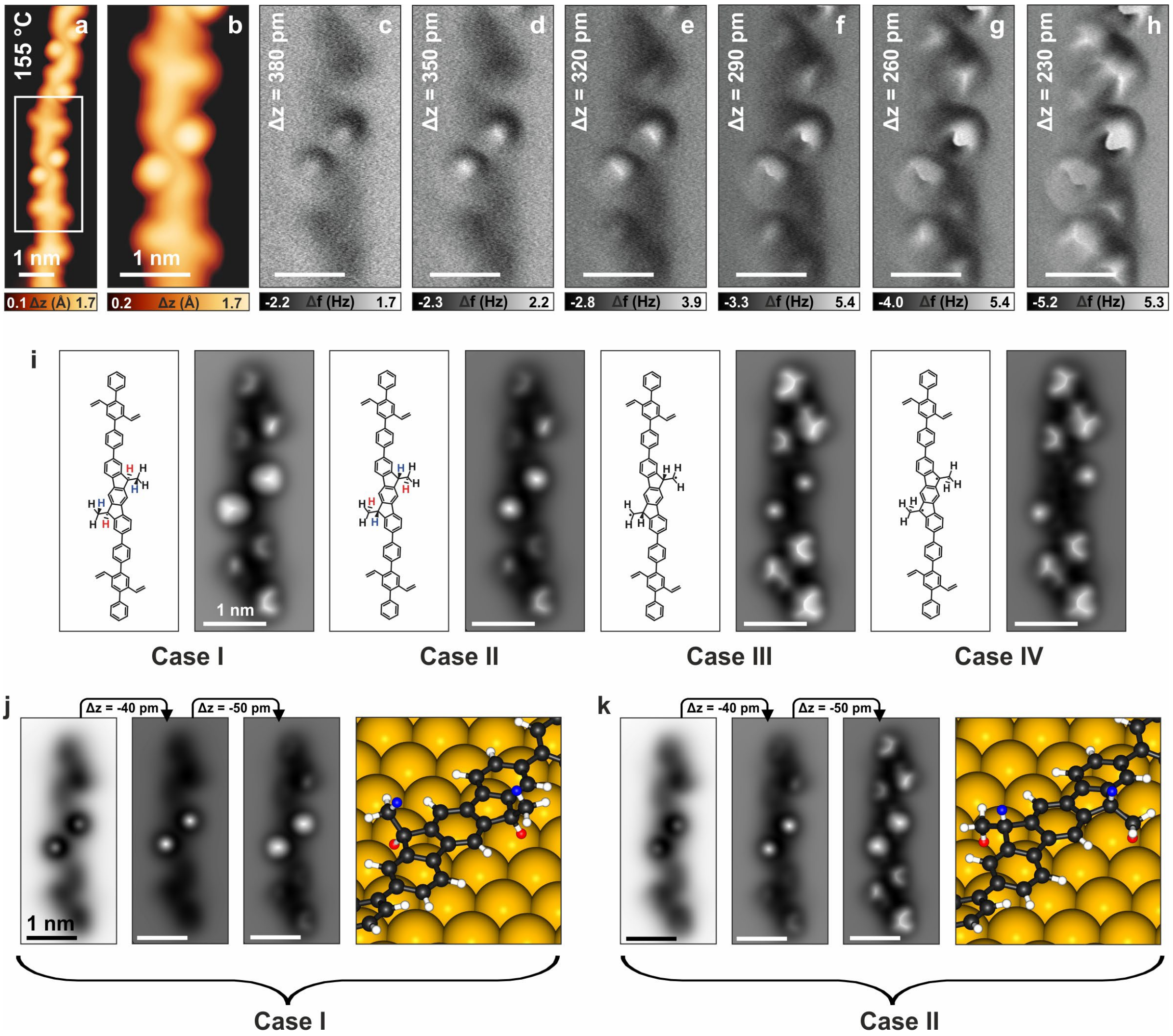

Supplementary Fig. 4 | Identification of the intermediate 3. a, STM image of a polymer segment after annealing the sample to 155 °C and **b**, zoom in of the highlighted part. **c-h**, Constant-height frequency shift nc-AFM images with a CO functionalized tip of the segment in **b** at decreasing tip-surface distance. **i**, Simulated nc-AFM images of four oligomers (Case I-IV) where two units **2** sandwich a unit where the vinyl groups have cyclized to form a five-membered ring, with various numbers and orientations of the hydrogen atoms. In the experimental nc-AFM images, the two dot-like features appear significantly higher than the surrounding vinyl groups. This is only reproduced by Case I and II, where no hydrogen loss has occurred yet, while Case III and IV can be discarded. **j,k**, To assess the orientation of the hydrogen atom and methyl group linked to the sp^3 carbon atom at the apex of a five-membered ring, we studied height-dependent simulated nc-AFM images. Reducing the tip-surface distance by 90 pm from a distance where the dot-like features start to appear in the image shows still faint vinyl features in Case I, but comparable signal intensity for vinyls and dots in Case II. Comparison with the experimental nc-AFM images (**c,f**) suggests that Case I better describes this intermediate structure, with the hydrogen and methyl group at the apex of five-membered rings pointing toward and away from the surface, respectively. The oligomer of Case I is energetically more stable than that of Case II (the total energy difference between the two geometries on Au(111) calculated by DFT is –0.3 eV), further supporting our assignment. Scanning parameters: **a,b**, I_t=30 pA, V_b=–0.3 V; **c-h**, Δz above the STM set point (I_t=100 pA, V_b=–5 mV) is given for each image. Temperature in **a** indicates the sample annealing temperature, while the imaging was performed at 4.7 K.

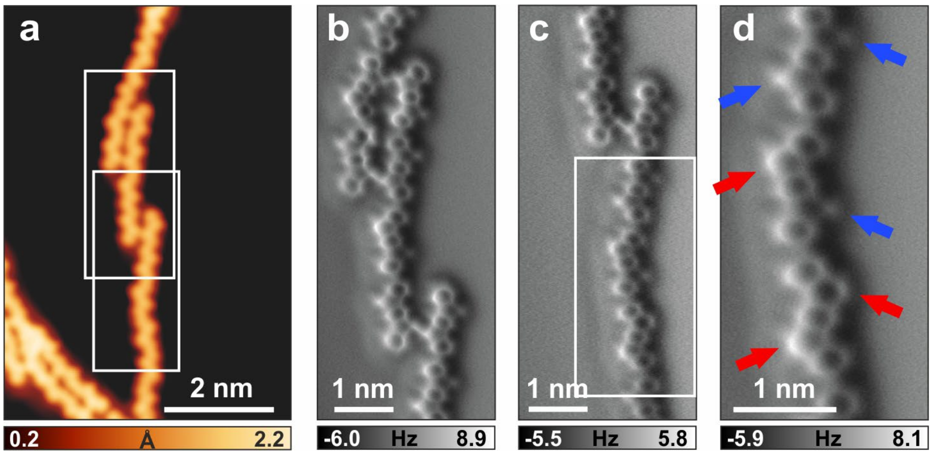

Supplementary Fig. 5 | Pentagon to hexagon transformation at one side of the repeating unit only. a, STM image of polymer segments acquired after annealing the sample to 380 °C (I_t=100 pA, V_b=–0.1 V). **b,c**, Constant-height frequency shift nc-AFM image with a CO functionalized tip of the segments highlighted in **a**. **d,** nc-AFM image of the segment highlighted in **c**. Blu and red arrows indicate the molecular sites that are converted into pentagons and hexagons (i.e. representative of structure **4** and **5**), respectively.

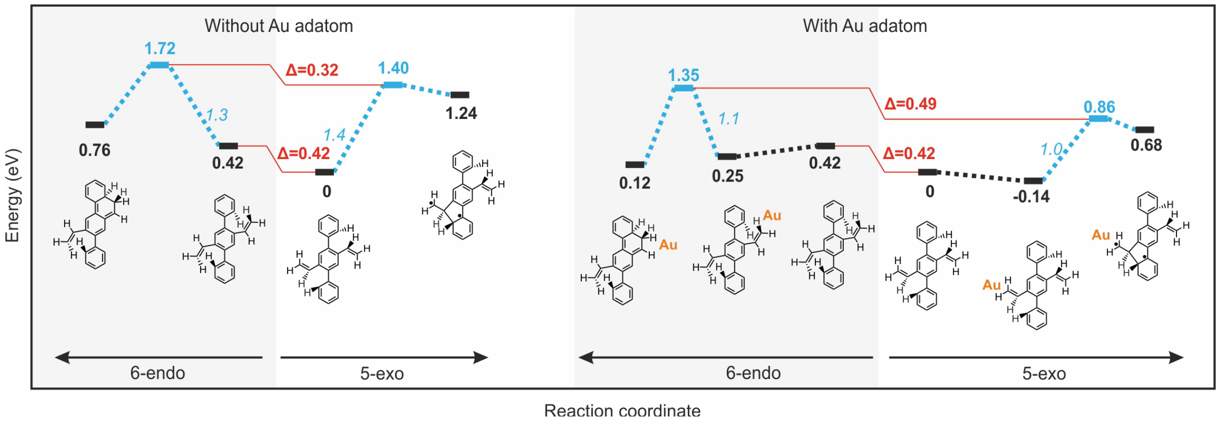

Supplementary Fig. 6 | Comparison between the initial cyclization into pentagon or hexagon in absence (left) and in presence (right) of a gold adatom next to the reacting group. Black bars and energy values represent the total energy (black bold) of the DFT-optimized geometries of the various structures on Au(111). Equilibrium structures are reported for each of these states. Cyan bars and energy values refer to the transition states (cyan bold) and energy barriers (cyan italic) of each reaction step. The energy barriers within this graph have been obtained using a series of constrained geometry optimizations (see section 3 below). The orange labels indicate gold adatoms close to the reacting groups. Grey background distinguishes the six-membered ring formation, with reaction coordinate increasing from right to left.

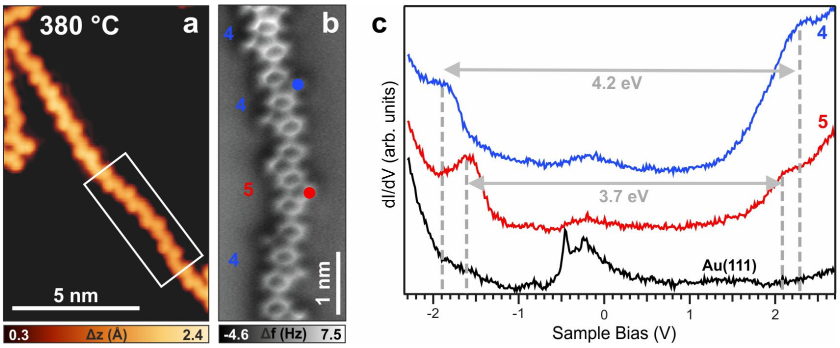

Supplementary Fig. 7 | STS measurements on units of type 4 and 5. a, STM image of a polymer segment acquired after annealing the sample to 380 °C, where repeating units of type **4** and **5** coexist. **b**, Constant-height frequency shift nc-AFM image with a CO functionalized tip of the segment highlighted in **a**. **c**, dI/dV spectra acquired at specific locations of the polymer, indicated with the blue and red dots in **b** and corresponding to repeat units with structure **4** and **5**. The black curve is a reference spectrum taken on the bare Au(111) surface. Units of type **5** exhibit a decreased band gap (3.7 eV) compared to **4** (4.2 eV), most likely due to an increased conjugation within the building block. Temperature in **a** indicates the sample annealing temperature, while the imaging was performed at 4.7 K.

3. Energy barrier estimation

3.1 Constrained geometry optimizations and nudged elastic band calculations

The energy barriers reported in this work are obtained using the climbing image nudged elastic band (CI-NEB) method,[6] from initial guesses derived from series of constrained geometry optimizations (CGO), as described below.

In order to characterize the reactions occurring on the surface, as a first step we identified metastable intermediate states matching the experimentally observed ones. From a starting geometry, we established a chemically valid reaction path using a collective variable (CV), such as an interatomic distance. We slowly adjusted this variable's value (incrementing by 0.05 Å on interatomic distances), consistently optimizing the system's geometry while keeping the CV constrained to its actual value until reaching a new minimum. During this process, we allowed all other degrees of freedom to relax until reaching a minimum energy state. This iterative procedure continued until a new local energy minimum was attained. In each optimization step, all atomic positions were relaxed until the forces acting on the atoms were below 10^{-4} atomic units. After each CGO series, the resulting "end geometry" was re-optimized to identify the most stable local minimum, which served as the starting point for subsequent steps. The highest point along this path approximated a transition state. To refine our estimation of the transition state's energy, we performed CI-NEB calculations (with a convergence threshold on the barrier set at 0.02 eV) on the initial minimum energy paths (MEPs) obtained from CGOs. We utilized 14 replicas for each NEB calculation.

For the ring rearrangement from **IV** to **V** we selected as CV the distance between the two C atoms highlighted in red in Supplementary Fig. 8. In this case, without the inclusion of a gold adatom, a barrier higher than 3 eV for the C-C bond breaking was found. On the other hand, the gold adatom reduces the barrier to 2.30 eV. We think that before a complete opening of the C-C bond, hydrogen migration occurs – as depicted in Fig. 5i in the main text – which initiates the ring closure to **V**, allowing for an even smaller activation barrier. A proper investigation of this concerted mechanism would require advanced techniques such as metadynamics[7] that

would be computationally prohibitive within DFT for our system thus going beyond the scope of the present study.

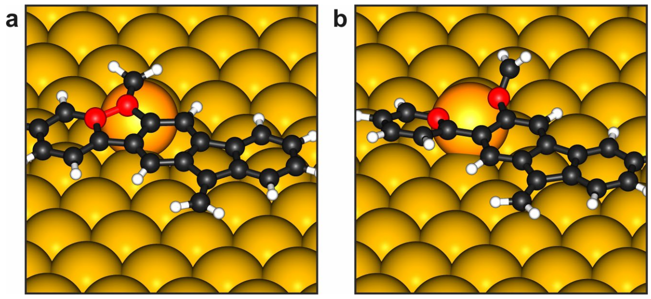

Supplementary Fig. 8 | DFT-optimized geometries during the C-C bond scission that triggers the final ring rearrangement. a, Equilibrium geometry of structure **IV**. The C atoms undergoing bond breaking are highlighted in red, while the Au adatom that allows to reduce the reaction barrier is highlighted in orange. By increasing the target distance between the two atoms, the state of highest energy is found at 2.30 eV (panel **b**). We hypothesize that, before reaching the configuration shown in panel **b**, a hydrogen migration occurs – as depicted in Fig. 5i in the main text – inducing the ring closure into species **V**.

3.2 General considerations

Here, we address the comparison between energy barriers determined from CGOs and CI-NEB calculations. Within standard transition state theory, a series of CGOs results into a MEP, and for each value of the CV there is a well-defined value for the energy of the system. Moreover, the maximum in between two minima along this path is, by definition, a transition state.[8] It's important to note that the method of slowly varying a constraint is common in molecular dynamics simulations aimed at performing thermodynamic integration, a method in use for decades that refers back to the coupling parameter method of Kirkwood.[9,10] In our scenario we perform a zero-temperature investigation and, due to discretization, achieving the exact transition state becomes improbable, resulting in either 'overshooting' or 'undershooting' it. This is why, if higher accuracy must be met, it could be necessary to refine the identified MEP with a method such as NEB. However, for a qualitative understanding of a chemical reaction, the computationally intensive NEB calculation might be bypassed, and the energy landscape derived from CGOs could suffice. To support this assertion, we report in Supplementary Fig. 9 the energy barriers presented in the main text – obtained via the NEB method – along with those obtained from the series of CGOs. The observed differences between values obtained from these methods consistently remain below 0.08 eV.

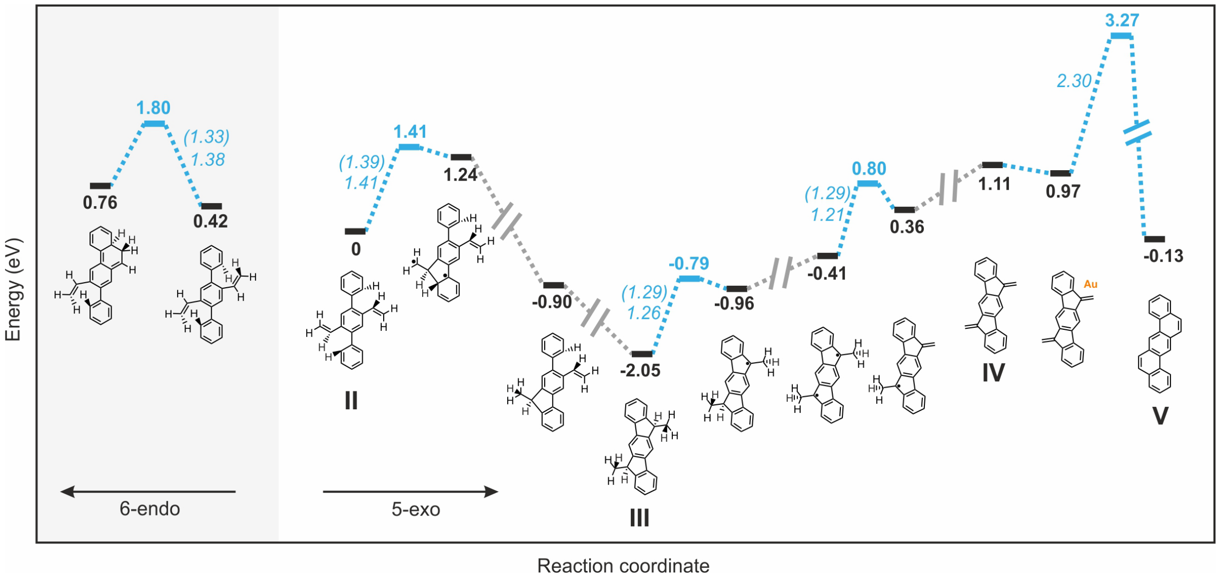

Supplementary Fig. 9 | Comparison between the energy barriers computed with different methods. Energy landscape reported in Fig. 6 in the main text (see the description reported therein for the details). Cyan italic values refer to the activation energies of each reaction step, obtained by the climbing image nudged elastic band (CI-NEB) method. The values in parentheses indicate the energy barriers derived from the series of constrained geometry optimizations (CGOs).

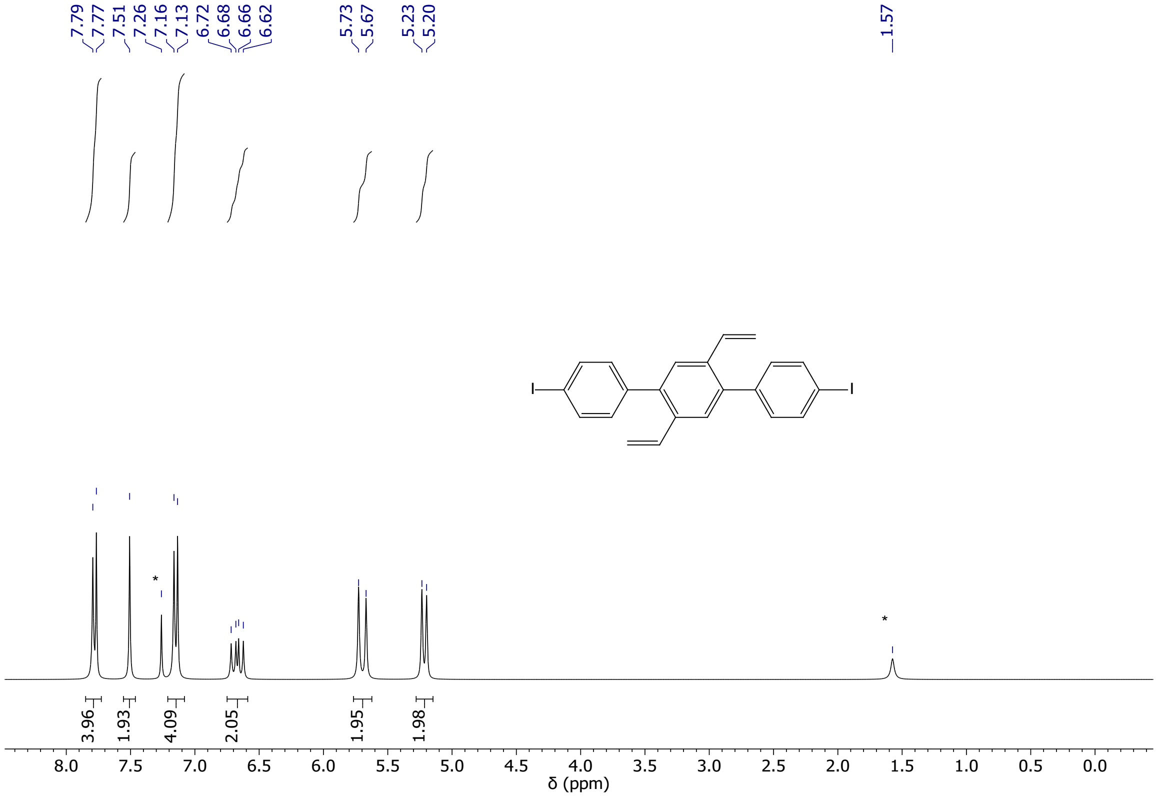

Supplementary Fig. 10 | NMR spectrum. ^{1}H NMR spectrum of precursor **1** in $CDCl_3$ (300 MHz, 298 K). The solvent peaks were marked with asterisks.

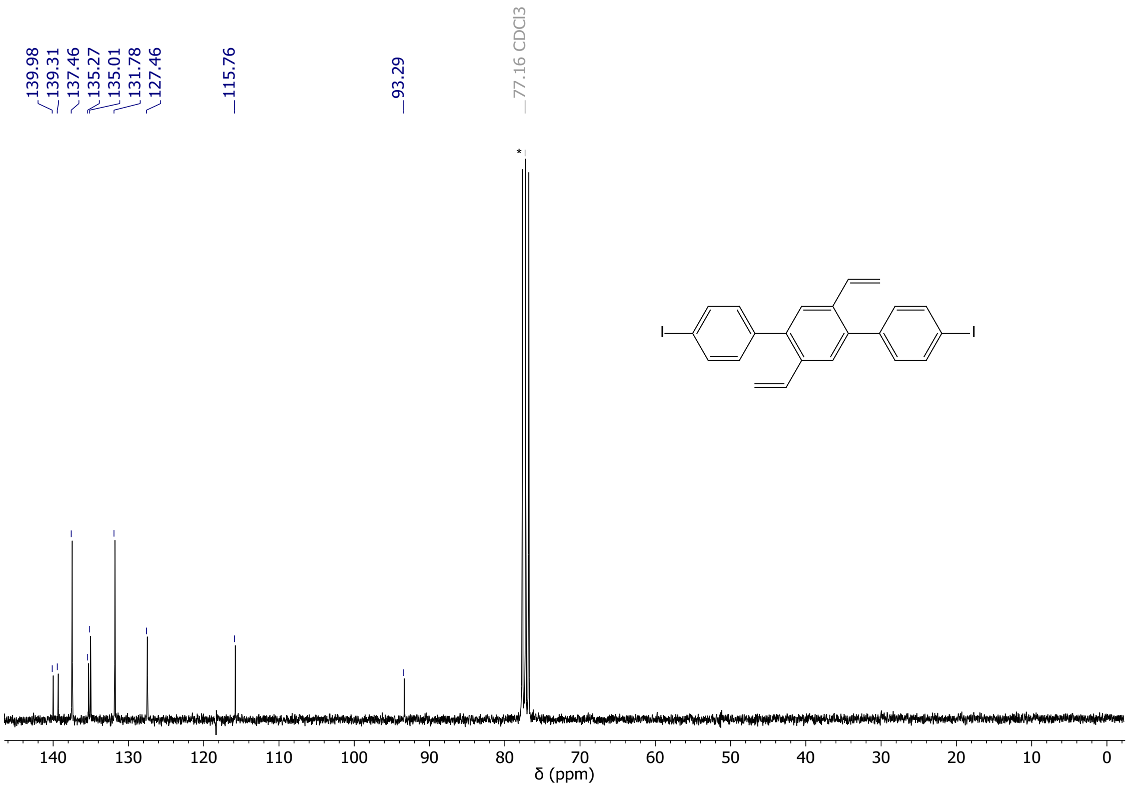

Supplementary Fig. 11 | NMR spectrum. ^{13}C NMR spectrum of precursor **1** in $CDCl_3$ (75 MHz, 298 K). The solvent peaks were marked with asterisks.

References

(1) Bonifacio, M. C.; Robertson, C. R.; Jung, J.-Y.; King, B. T. Polycyclic Aromatic Hydrocarbons by Ring-Closing Metathesis. *J. Org. Chem.* **2005**, *70* (21), 8522–8526. https://doi.org/10.1021/jo051418o.

(2) Vermeulen, N. A.; Karagiaridi, O.; Sarjeant, A. A.; Stern, C. L.; Hupp, J. T.; Farha, O. K.; Stoddart, J. F. Aromatizing Olefin Metathesis by Ligand Isolation inside a Metal–Organic Framework. *J. Am. Chem. Soc.* **2013**, *135* (40), 14916–14919. https://doi.org/10.1021/ja407333q.

(3) Zhao, Z.; Britt, L. H.; Murphy, G. K. Oxidative, Iodoarene-Catalyzed Intramolecular Alkene Arylation for the Synthesis of Polycyclic Aromatic Hydrocarbons. *Chem. – Eur. J.* **2018**, *24* (64), 17002–17005. https://doi.org/10.1002/chem.201804786.

(4) Matsuyama, H.; Zhang, X.; Terada, M.; Jin, T. Construction of Alkylidene Fluorene Scaffolds Using Pd-Catalyzed Direct Arene/Alkene Coupling Strategy. *Org. Lett.* **2023**, *25* (5), 800–804. https://doi.org/10.1021/acs.orglett.2c04307.

(5) Horgan, S.; Morgan, D.; Orchin, M. The Photochemistry of 2-Vinylbiphenyl And. *J. Org. Chem.* **1973**, *38* (21), 3801–3803. https://doi.org/10.1021/jo00961a600.

(6) Henkelman, G.; Uberuaga, B. P.; Jónsson, H. A Climbing Image Nudged Elastic Band Method for Finding Saddle Points and Minimum Energy Paths. *J. Chem. Phys.* **2000**, *113* (22), 9901–9904. https://doi.org/10.1063/1.1329672.

(7) Bussi, G.; Laio, A. Using Metadynamics to Explore Complex Free-Energy Landscapes. *Nat. Rev. Phys.* **2020**, *2* (4), 200–212. https://doi.org/10.1038/s42254-020-0153-0.

(8) Jensen, F. *Introduction to Computational Chemistry*; John Wiley & Sons, 2017.

(9) Kirkwood, J. G. Statistical Mechanics of Fluid Mixtures. *J. Chem. Phys.* **2004**, *3* (5), 300–313. https://doi.org/10.1063/1.1749657.

(10) Frenkel, D.; Smit, B. *Understanding Molecular Simulation: From Algorithms to Applications*; Elsevier, 2023.
